# William Budd: Revolutionizing Epidemiology and Public Health in the 19th Century

**DOI:** 10.7759/cureus.76278

**Published:** 2024-12-23

**Authors:** Sheuli Paul, Shradha Salunkhe, Shailaja V Mane, Shivani Kale, Sauvik Paul

**Affiliations:** 1 Paediatrics, Dr. D. Y. Patil Medical College, Hospital and Research Centre, Dr. D. Y. Patil Vidyapeeth (Deemed to Be University), Pune, IND; 2 General Medicine, Sikkim Manipal Institute of Medical Sciences and Central Referral Hospital, Gangtok, IND

**Keywords:** 19th century medicine, epidemiology, germ theory, historical vignette, preventive medicine, public health disease, sanitation reform, transmission, typhoid fever, william budd

## Abstract

William Budd (1811-1880) was a pioneering British physician whose work on infectious diseases, particularly typhoid fever, significantly advanced the understanding of epidemiology and public health in the 19th century. This review examines Budd's life, medical career, and groundbreaking contributions to the study of infectious diseases, focusing on his influential research into the transmission of typhoid fever, advocacy for public health reforms, and lasting legacy in epidemiology. Through meticulous research and innovative thinking, Budd laid the groundwork for modern approaches to disease control and prevention, cementing his place as a key figure in medical history.

## Introduction and background

Early life and education

William Budd was born in the small market town of North Tawton, Devon, on September 14, 1811. He was one of 10 children born into a family with strong ties to the medical profession. His father, Samuel Budd, was an esteemed local doctor, and several of Budd’s brothers also chose to practice medicine. From an early age, young William was exposed to the practice of medicine, living in a household with his accomplished brothers. It could not have helped but spark his interest in the field, which later became a major area of study for him while at undergraduate school. Growing up in this environment also provided him with practical insights and inspiration, allowing him to navigate real-life medical scenarios with ease [1].

Budd first attended school locally when he was a boy and demonstrated aptitude in science and natural history. His academic faculties ultimately allowed him to do so when he went on for formal medical training. At the tender age of 16, William embarked on a formative medical journey in Paris, studying for four transformative years. Under the mentorship of the renowned clinician and epidemiologist Pierre Charles Louis, he honed his skills in distinguishing the nuances between typhoid and typhus. While William's ability to recognize these diseases in living patients was developing, his expertise truly blossomed through meticulous post-mortem examinations, where he meticulously observed the inflamed and ulcerated Peyer's patches that characterized these devastating conditions. For his further medical education in the advanced realm, he enlisted at the esteemed University of Edinburgh, which was one of the best medical centers in Europe during that era. Edinburgh University was the place that used to distinguish itself by being rigorous and evidence-based, where Budd learned a lot about epidemiology and public health [2]. At Edinburgh, Budd had access to the most recent medical theories and practices available. He was taught by leading physicians and scientists of the time such as Dr. Robert Christison, an expert in toxicology, and Dr. James Syme, who was at the forefront of surgical practice [3,4]. Through this immersion in the cutting-edge science of medicine, Budd quickly acquired a fundamental understanding and integration into clinical care concepts as well as research. After graduating in medicine (MD) in 1838, where he earned a gold medal for his study on acute rheumatism, he spent additional time on the European medical circuit. He nearly died from typhoid fever while working at a naval hospital, HMS Dreadnought [5].

Early career and influences

Following a period of medical study, Budd returned to Devon where he started his practice as a physician. He went into practice with his father first, which provided him with a lot of experience in treating all kinds of medical conditions. Based in rural Devon, Budd came face to face with the unsparing facts of 19th-century medical practices and had to overcome the fact that infectious diseases are still running riot combined with very few methods available for treating them. Those early experiences deeply influenced his thinking about health care and, later, public health. It was during this time that Budd began to pay closer attention to the epidemiology of diseases and how infectious agents might be transmitted. Of all the diseases he encountered, Budd was particularly struck by the impact of an illness, widely known as typhoid fever due to certain similarities with typhus, although the two conditions were occasionally conflated, on rural communities in places like Devon. Typhoid fever claimed many lives in the 19th century due to its high-grade fevers, abdominal pains, and severe diarrhea, but it remained a relatively poorly understood disease [6]. The causative bacterium, Salmonella typhi, was eventually identified in 1880 by Karl Joseph Eberth [7]. In 1836, William Gerhard of Philadelphia conducted a groundbreaking study that definitively differentiated typhus and typhoid fevers. Through postmortem examinations of six patients, he observed the remarkable absence of ulceration in Peyer's patches, a finding that starkly contrasted with the typical pathological features of typhoid fever [8]. Sir William Jenner observed similar findings and published a monograph in 1850, titled, "On the identity or non-identity of typhoid and typhus fevers," which differentiated the two conditions [9].

**Figure 1:**
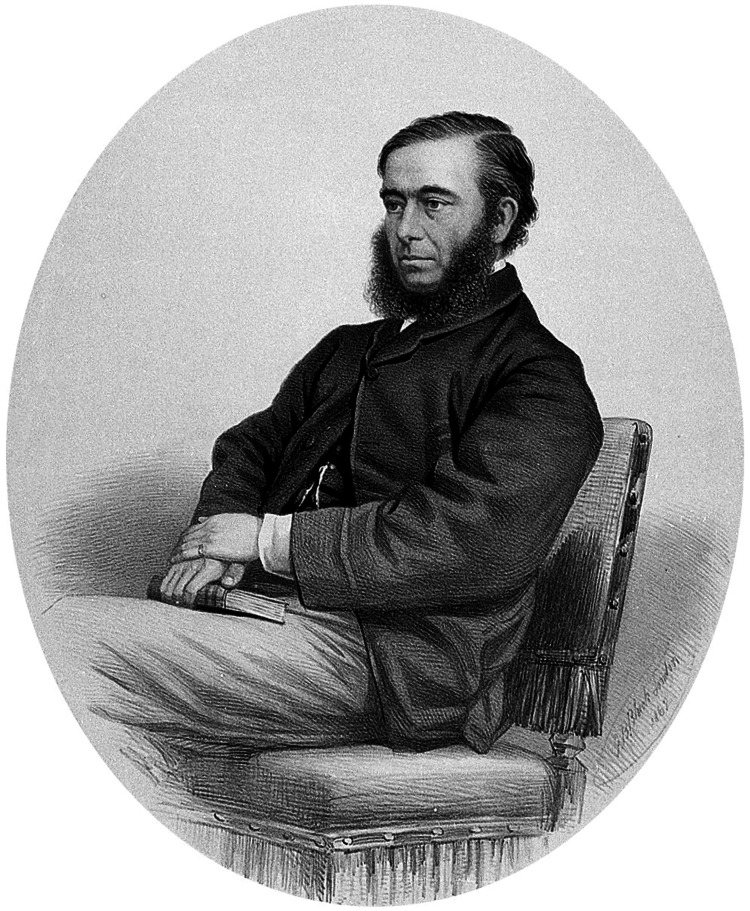
Portrait of William Budd from an original lithograph published by A.B. Black, 1862, in the Royal Society of Medicine Credit: Unknown Author Source: Wellcome Collection, London [5]. This file is licensed under the Creative Commons Attribution 4.0 International license. (CC BY 4.0) [11].

Budd was further motivated to know all he could about infectious diseases by the discoveries of other seminal physicians and scientists. Perhaps the person who most shaped Budd's thought was John Snow, a London doctor widely viewed as the father of modern epidemiology. His pioneering work on cholera proved its true name to be the result of contamination in water as opposed to the miasma theory that was then prevalent. This idea was that diseases were spread by disease-causing agents in the environment, which included noisome vapors from putrefying organic materials. Snow's method laid the foundation for a new theory of disease: that contagion is primarily caused by particles passed from one person to another, and those particles generally could be isolated in some way. Budd was also influenced by the broader scientific advances of his time, particularly the emerging field of microbiology. Despite not being fully developed, the germ theory of disease, which proposed that germs were the cause of many diseases, was starting to gain support from the research of experts such as Louis Pasteur and Robert Koch. Budd's own meticulous observations and rigorous research would soon expand the understanding of this disease's epidemiology (Figure 1) [10].

## Review

Typhoid fever and the germ theory of disease

One of the most significant contributions William Budd made to medicine was his study of typhoid fever. In the mid-19th century, physicians lacked a clear understanding of the precise causes of typhoid fever, and this form of enteric illness was often dismissed as a vague, inflammatory condition. Existing medical theories, such as miasma theory, could not satisfactorily explain how typhoid was transmitted from person to person. Budd's meticulous research aimed to address this gap in scientific knowledge. Budd had become intrigued with typhoid fever after a protracted succession of severe outbreaks in the Devon area during the 1840s and 1850s. Through careful observation and diligent epidemiology data collection, Budd began to observe the way disease is transmitted in households and communities. He noticed that typhoid in general struck many members of the same household and appeared to correlate with certain water supplies. Based on these observations, he concluded that typhoid was not due to miasmas or polluted air, but rather by a specific transmissible pathogen [12].

Budd hypothesized that the agent responsible for typhoid fever was present in the excreta of infected individuals and that it could contaminate water supplies, leading to new cases of the disease. This marked a stark departure from miasma theory and was contemporary with the rise of germ theory. To test his hypothesis, Dr. Budd embarked on an exhaustive series of epidemiological studies in which he followed patients from the first sign of infection and traced each to its source during outbreaks. He made use of the maps to keep an eye on how the disease was spreading and he even tracked down a few common sources from where the water would get contaminated. In one well-known case study, Budd investigated an outbreak of typhoid fever in the village of Cowbridge and located a particular well from which people drank to be causing infection. He proved that the well had been polluted with sewage out of a nearby cesspool, which contained the excreta of a typhoid patient. Budd's work proved typhoid was a water-borne disease by fecal contamination. Budd had written a groundbreaking paper in *The Lancet*, one of the foremost medical journals, in 1859. In this paper, Budd described his view that typhoid was due to a "poison" (now recognized as Salmonella typhi) excreted by patients and spread through water and food. He maintained that the disease could be eliminated by reducing or eliminating their sources of origin, most importantly through the safe disposal of human waste and protection of water from fecal contamination [10]. The work of Budd on typhoid was an important early demonstration that specific diseases are caused by discrete living organisms and laid the foundations for germ theory, which would later be proposed in full form through experimentally better-supported findings by Louis Pasteur (1822-1895) and Robert Koch. While he lacked the means to see the organism responsible for typhoid fever, his thorough epidemiologic and purely deductive methods offered compelling but circumstantial evidence that pointed toward a pathogen with a discrete identity. This helped change the understanding of the medical community from miasmas to scientific evidence; teaching that diseases spread via contagion [13].

Public health advocacy

In addition to being an innovative scientist, he became a tireless champion for improvements in public health. He realized that controlling the transmission of infectious disease meant more than patient care; it required a transformation in public health methodology and structure. Budd and others were only too aware of the woeful state of sanitation in much of England, so bad that rural areas often did not benefit from appropriate sewage disposal or clean water supplies. Budd believed public health to be a collective responsibility and that medical professionals like himself had an obligation to educate the general population as well as lawmakers of just how vital proper sanitation and cleanliness were. He was an early innovator in what today we would call preventive medicine, advocating a determination to take down the roots of illness rather than merely removing its flowers [5].

Budd's key epidemiological contributions included insisting on proper sanitation practices, such as ensuring clean sewage discharge and maintaining separation between drinking water and waste disposal systems. He knew that typhoid fever and other infectious diseases were spreading through contaminated water, and he insisted on making the protection of water supplies central to public health. Through his writings and lectures, Budd argued for the installation of sewage treatment systems to prevent drinking water from being polluted. He also supported the use of disinfectants to purify sewage and for clean, unpolluted water systems so that citizens could have access to clean, uncontaminated water. His research was fundamental to numerous reforms in public health that finally got off the ground during the years following 1848 due largely to repeated epidemics of typhoid fever and cholera [14]. In addition to his work on sanitation, Budd was also a strong advocate for the isolation of infectious disease patients to prevent the spread of disease within hospitals and communities. He appreciated the value of adopting public health measures, such as quarantine and laundry disinfection, to prevent diseases like typhoid fever. The practices that he recommended for isolation and disinfection eventually became standard public health protocols that are still used today to help fight the spread of infectious diseases.

Impact on modern public health and epidemiology

Budd's emphasis on the importance of sanitation and clean water in preventing disease laid the groundwork for the development of modern public health systems. Many of his recommendations for sewage treatment and the protection of water supplies have since become standard public health practices throughout much of the world. The principles that Budd espoused - clean water, safe disposal of excretions, and isolating infected patients from the rest of society to prevent them from passing it on - are now central tenets in public health campaigns against disease [15].

In addition, Budd's methods of investigation on disease outbreaks then, which were characterized by meticulous observation and recording as well as detailed analysis, paved the way for the methods used in modern epidemiology. The way he used maps to track the spread of disease and identify new points of infection foreshadowed techniques that epidemiologists still employ today, such as mapping disease patterns, diagnosing and prognosing infectious diseases, and evaluating intervention outcomes. Budd's suggestions were largely implemented in the late 19th and early 20th centuries; such recommendations found particular favor within developing cities confronting epidemics (e.g., cholera and typhoid) and have led to substantial expenditures on sanitation facilities for water, waste, sewage, etc. Budd's work produced critical groundwork for the establishment of modern public health systems, which focus on trying to prevent diseases through surveillance, reduced exposure, and education about prevention [16].

Later life and legacy

William Budd, in his later career, went on to further develop these concepts and the actions related to public health. He wrote numerous articles and books confirming his beliefs about the mechanisms of transmission and prevention of disease. In 1873, he published "Typhoid Fever: Its Nature, Mode of Spreading and Prevention," a book that summed up his research on the disease as well as provided recommendations for public health. This book was widely read and respected in the medical community and contributed to the growing acceptance of the germ theory of disease [17].

Budd had an influence beyond his immediate field of work. Budd's influence extended far beyond his own time. His public health perspective recognized the environmental factors contributing to disease causation, and his ecological approach to prevention aligned with our modern understanding. Through his work, the value of systematically observing and quantifying patterns was brought to the forefront, informing public health practice. Budd's belief in the fundamental importance of sanitation and clean water to disease prevention inspired many of the improvements that elevated public health standards, including the widespread implementation of sewage treatment and purified piped water that we continue to rely on today [18]. Despite the significance of his contributions, Budd did not achieve the same level of fame as some of his contemporaries, such as John Snow or Louis Pasteur. His work was in part eclipsed by more dramatic findings of the microbiologists, who pinpointed germs that caused diseases - such as typhoid fever. In recent years, historians of medicine have acknowledged Budd as one of the true fathers of modern epidemiology and public health [19,20].

William Budd passed away on January 9, 1880, at the age of 68. William Budd's enduring legacy continues to inspire today's medical and public health professionals. His unwavering commitment to rigorous scientific investigation and tireless advocacy for public health measures has cemented his status as a pivotal figure in the history of medicine. Budd's work underscores the vital importance of looking beyond surface-level symptoms to uncover the fundamental drivers of disease and of proactively implementing preventive strategies. His groundbreaking contributions to the understanding of infectious diseases and the advancement of public health practices have left an indelible mark, solidifying his place as a true pioneer in the field [21].

## Conclusions

William Budd was a medical pioneer whose studies into typhoid fever and the transmission of diseases on entire communities have helped establish the modern frameworks we use in epidemiology and public health. His careful examination, path-breaking theories, and zest for advocacy significantly revolutionized the field of knowledge that informs how we understand infectious diseases spread and how they can be controlled. The way in which Budd was able to improve sanitation, safeguard water supplies, and disrupt the spread of contagious diseases are key aspects of his legacy, which remain visible today. William Budd may not be as well-known today as his contemporaries, but some of his work in medicine and public health remains among the foundational contributions that continue to shape current-day prevention practices.
